# Supplementary material for: C-Reactive Protein and White Blood Cell Count in Non-Infective Acute Ischemic Stroke Patients Treated with Intravenous Thrombolysis
Source: J Clin Med. 2021 Apr 10;10(8):1610. doi: 10.3390/jcm10081610 (PMC8069454; doi:10.3390/jcm10081610)
Supplement: Supplementary file 1 [file jcm-10-01610-s001.pdf]

Supplemental Table S1. Baseline clinical characteristics of the patients based on white blood cell count (WBC) quartiles.

	**Q1**<br>**WBC < 6.40 × 10⁹/L**<br>***n* = 40**	**Q2**<br>**WBC 6.40–7.75 × 10⁹/L**<br>***n* = 39**	**Q3**<br>**WBC 7.76–9.60×10⁹/L**<br>***n* = 39**	**Q4**<br>**WBC ≥ 9.61 × 10⁹/L**<br>***n* = 40**	***p*-value**
Age (years)	72 (68-83)	75 (68-83)	72 (64-80)	67 (59-80)	0.145
Women, *n* (%)	22 (55.0)	25 (64.1)	17 (43.6)	20 (50.0)	0.314
BMI (kg/m^2)	27.4 (24.1–29.4)	25.7 (22.5–29.3)	25.4 (24.2–27.6)	27.3 (24.8–29.6)	0.201
Hypertension, *n* (%)	34 (85.0)	31 (79.5)	29 (74.4)	24 (85.0)	0.571
Hypercholesterolemia, *n* (%)	11 (27.5)	11 (28.2)	13 (33.3)	13 (32.5)	0.921
Diabetes mellitus, *n* (%)	14 (35.0)	7 (18.0)	10 (25.6)	11 (27.5)	0.395
Smoking, *n* (%)	3 (7.5)	6 (15.4)	7 (18.0)	7 (17.5)	0.520
Ischemic heart disease, *n* (%)	9 (22.5)	7 (18.0)	8 (20.5)	11 (27.5)	0.771
Atrial fibrillation, *n* (%)	15 (37.5)	13 (33.3)	9 (23.1)	7 (17.5)	0.171
Previous stroke, *n* (%)	7 (17.5)	7 (18.0)	7 (18.0)	7 (17.5)	1.000
mRS score before stroke > 0	5 (12.5)	0 (0.0)	2 (5.1)	4 (10.0)	0.133
Stroke etiology, *n* (%)					
- large-vessel disease	1 (2.5)	4 (10.3)	10 (25.6)	8 (20.0)	
- small-vessel disease	0 (0.0)	2 (5.1)	0 (0.0)	0 (0.0)	
- cardioembolic	17 (42.5)	12 (30.8)	10 (25.6)	11 (27.5)	0.089
- other	21 (52.5)	19 (48.7)	19 (48.7)	20 (50.0)	
- undetermined	1 (2.5)	2 (5.1)	0 (0.0)	1 (2.5)	
Stroke etiology, *n* (%)<br>- large-vessel disease	1 (2.5)	4 (10.3)	10 (25.6)	8 (20.0)	0.018
Mechanical thrombectomy, *n* (%)	17 (42.5)	9 (23.1)	8 (20.5)	13 (32.5)	0.128
Time from stroke onset to thrombolysis (min)	119 (85–190)	106 (75–145)	125 (82–170)	138 (89–190)	0.559
NIHSS score on admission	11.9 ± 6.8	11.9 ± 6.7	9.8 ± 6.8	12.5 ± 6.3	0.278
NIHSS score after r-tPA	6.4 ± 8.7	6.6 ± 6.1	6.4 ± 6.2	8.7 ± 9.0	0.329

Post-IVT hemorrhagic brain complications, *n* (%)					
- no complication	31 (77.5)	31 (79.5)	36 (92.3)	32 (80.0)	
- HI type 1	5 (12.5)	3 (7.7)	1 (2.6)	2 (5.0)	
- HI type 2	2 (5.0)	3 (7.7)	0 (0.0)	3 (7.50	0.677
- PH type 1	1 (2.5)	2 (5.1)	1 (2.6)	1 (2.5)	
- PH type 2	1 (2.5)	0 (0.0)	1 (2.6)	2 (5.0)	
Maximal SBP within 24 hours after r-tPA (mmHg)	145 (131–157)	147 (135–168)	147 (123–170)	140 (130–152)	0.419
Maximal DBP within 24 hours after r-tPA (mmHg)	80 (70–90)	80 (72–85)	80 (72-90)	79 (67–88)	0.330
Fasting glucose (mmol/L)	6.4 (5.7–7.7)	6.5 (5.3–7.9)	6.2 (5.5–7.1)	7.0 (5.7–9.0)	0.858
Creatinine (µmol/L)	83 (71–97)	76 (60–93)	74 (64–86)	80 (72–98)	0.239
CRP (mg/L)	4.3 (1.8–6.6)	3.8 (1.8–6.7)	3.1 (1.8–5.4)	5.9 (3.1–10.1)	0.107

Abbreviations, see Table 1. Q1–Q4 denotes four groups according to the quartile of WBC (x10^9/L).

Supplemental Table S2. Predictors of the CRP ≥ 8.65 mg/L (fifth quintile).

**CRP ≥ 8.65 ng/L**	**Univariate**			**Multivariate**		
	HR	95% CI	*p*-value	HR	95% CI	*p*-value
Age (per 1 year)	1.02	0.99–1.06	0.153	-	-	-
Sex (female)	2.13	0.93–4.89	0.073	-	-	-
BMI (per 1 unit)	1.11	1.01–1.22	0.033	-	-	-
Hypertension	8.89	1.16–67.9	0.036	8.12	1.05–62.9	0.045
NIHSS score on admission	1.09	1.03–1.16	0.006	1.09	1.02-1.16	0.011

Abbreviations, see Supplemental Table 1, 2.

Supplemental Table S3. Predictors of white blood cells count < 6.4x10^9/L (first quartile).

WBC < 6.4x10^9/L (Q1)	Univariate			Multivariate		
	HR	95% CI	*p*-value	HR	95% CI	*p*-value
Age (per 1 year)	1.02	0.99–1.05	0.140	-	-	-
Sex (female)	1.24	0.61–2.54	0.547	-	-	-

BMI (per 1 unit)	1.02	0.93–1.11	0.721	-	-	-
Atrial fibrillation	1.93	0.91–4.11	0.086	-	-	-
Large vessel stroke	0.10	0.01–0.78	0.029	0.10	0.01–0.80	0.030
Mechanical thrombectomy	1.95	0.93–4.10	0.078	-	-	-

Abbreviations, see Supplemental Table 1, 2.
